# Cork: a strategic material

**DOI:** 10.3389/fchem.2014.00016

**Published:** 2014-04-11

**Authors:** Luís Gil

**Affiliations:** Unidade de Energia Solar, Laboratório Nacional de Energia e Geologia, I.P.Lisboa, Portugal

**Keywords:** cork, cork oak, sustainability, environment, CO_2_ uptake and CO_2_ fixation

Cork is a material whose applications have been known since Antiquity, especially in floating devices and as stopper for beverages, mainly wine, whose market, from the early twentieth century, had a massive expansion, particularly due to the development of several cork based agglomerates (Gil, 2011).

Cork is closely related to the maintenance of biodiversity, the heart of sustainable development, and the reduction of emissions and sequestration of CO_2_, aspects that, additionally to the environmental importance, are also economically very important (Gil, 2011). Other services such as the formation of the landscape, soil protection, regulation of hydrological cycle, are also very sound (Pereira, 2007). Cork regenerates after each stripping, and the cork tree survives the lost of an important quantity, often more than 50% of the total trunk and branches' surface. **The fact that corks are made of the bark harvested from living trees has lead environmentalists to encourage the use of cork over other, less natural, alternatives**.

Cork oak forests (known as “*montados*”) are a habitat for many animal and plant species. At a meeting of experts it was announced that the “*montado*” is integrated in one of the 34 “*hotspots*” of biodiversity worldwide, featuring a number of species per square meter even higher than the Amazon rainforest, usually stated as reference in this field. Cork oak is one of the best examples of real sustainability through the environmental, economic, and social functions within the various forest types, aspects currently placed on the agenda of the world public opinion. In addition to forest products' production and activities associated with the extraction of cork, other activities such as hunting, bee-keeping, livestock, harvesting of mushrooms, and herbs and medicinal plants, reflect a multi-functional system which has a great social and economic importance in regions where the cork oak grows (Pereira, 2007; Gil, 2010, 2011).

Significant reductions in emissions of greenhouse gases in the construction sector can be achieved through various measures for energy savings, an area where cork derivatives can have a very important role, particularly regarding the thermal performance of buildings, but not only. Cork used in floor coverings, wall coverings, and other decorative applications also contribute to this. Also considered are green building and sustainable materials which are concepts that are increasingly considered by engineers, architects and other technicians and even by consumers.

Almost all cork products can be recycled and the main advantage of this procedure is that this material incorporates carbon fixed by the cork tree that remains there during the lifetime of the products (long life products), thus increasing the delay of the emission of this carbon back to the atmosphere. Besides this if there is no more use after their useful life, cork products can be used in energy production, having a high calorific value and, when incinerated, the CO_2_ produced is equivalent to the one which was fixed in the material, what is commonly referred to as being “carbon neutral” (Gil, 2010, 2011).

In addition to this, the periodic extraction of cork oaks for cork production, produces between 250 and 400% more cork than they would produce if it they were not explored (when the bark is harvested the tree produces rapidly new bark for protection) increasing the fixation of CO_2_. Therefore, the consumption of cork products that leads to the exploitation of this material promotes the formation of more cork and thus more CO_2_ is sequestered, beyond the stated fact that such products are long-life products retaining the carbon during their useful life and being “carbon neutral” at the time of decomposition or energy use. Some studies refer that cork oak forests make a sequestration of until 5.7 ton CO_2_/ha/year. The 2.3 million ha of cork oak forests worldwide are seen as promoting the retention of about 14.4 million tonnes CO_2_/year. It should be noted also that according to data from a supplier 0.379 kg of CO_2_/kg of cork are emitted but each kg of final product is responsible for fixing 1.833 kg of CO_2_. Besides this, e.g., to produce 1000 cork stoppers 1.5 kg CO_2_ are emitted, but 14 kg of CO_2_ are emitted for the same amount of plastic stoppers or 37 kg CO_2_ for 1000 screwcaps (Gil, 1998, 2011; Pereira, 2007; Corticeira Amorim, 2008).

According to the Cork Portuguese Association Annuary 2012 (APCOR, 2013) the European countries where cork is produced, Portugal (715,922 ha, 100,000 ton/year), Spain (574,248 ha, 61,504 ton/year), France (65,228 ha, 5200 ton/year), and Italy (64,800 ha, 6161 ton/year), have more than 67% of the total cork forest area and produce more than 85% of the global cork production. The exports of cork products in 2011 where: Portugal—805 million euros; Spain—216 million euros; France—51 million euros; Italy—50 million euros, contributing for the economy of these countries. The exportation of cork products is worldwide. Several European countries are great importers of cork products, as for example, in 2011, Germany (104 million euros). The main country consuming natural cork stoppers is France, with over 100 million euros. France also leads the consumption of champagne cork stoppers, at 27 million euros, leaving Italy occupying the second place at 19 million euros.

Also according to this Annuary, the main sector for which cork products are destined is the wine bottling, which absorbs 70% of all production, followed by the building industry with 22%—which includes thermal, acoustic, and vibration insulation, wall and floor coverings, cubes, plates, sheets, strips, and even some products from the “other cork products” category.

Cork and wine have been “married” for thousands of years, first as cover of amphoras, pots and other wine vessels and in the last 300 years as stopper for bottled wine and this “marriage” has been evolving with the help of new technologies in both sides. The cork stopper is much more than just a simple seal and the future will identify it as an enological product, highly reliable. For example, a study concluded that the contact of wine with a cork granulate affects positively some wines, namely for wines not aged in oak barrels and mainly white wines, with the formation of a potent anti-tumoral agent and better organoleptic behavior (Gil et al., 2006).

Cork is a very versatile raw material which adopts different technological transformation processes giving rise to several products which can be used in different applications. The low thermal conductivity of this material combined with its reasonable compressive strength makes it an outstanding material for thermal insulation purposes mainly and advantageously when compressive loads exist. It also has anti-sliding properties which make it also excellent for floor coverings or handles. Today cork products are used for thermal insulation in refrigerators, cooling chambers, and rockets, acoustic insulation in submarines, theaters, and recording studios, seals and joints in woodwind instruments, combustion engines, and concrete constructions, and as energy-absorbing medium in floor coverings, shoes, and packaging, and as stoppers (Gil and Moiteiro, 2003).

So, cork is a material which has been used for the last 5 milleniums and it is still considered a strategic material used for a number of applications, from wine bottles to spacecrafts. Recent developments in cork research are changing this material from the classical cork-wine relationship to issues dealing with quality and environment, exploitation of cork industry wastes and new cork based materials. It was also demonstrated that after several decades of intensive use, insulation cork board (ICB) shows a thermal behavior better than the specified for ICB in relevant standards, being an example of the excellent life span of cork products (Gil and Silva, 2011).

In recent years a number of new cork based composite materials was developed. Some of these new applications of cork refer to, e.g., cork agglomerates as an ideal core material in lightweight structures (Silva et al., 2010), cork granulates for a bioremoval process of lead ions from effluents, new paints including cork particles for improvement of thermal and acoustic properties (Gil and Marreiros, 2011), only to name a few.

So, cork has clearly demonstrated that its application goes far beyond traditional products and this area of action is and will be fundamental for the sector's feasibility. As Sustainable Product Design is currently accepted as one of the most promising trends in “Sustainable Development,” cork can play a relevant role, because it is a natural, renewable, recyclable, and non-toxic resource, with exceptionally good environmental qualities, incorporating a high potential of innovative technological characteristics (Mestre and Gil, 2011).

Cork based products still lack the desired level of information and diffusion within stakeholders in the engineering, design, and selection of materials for construction, compared with other competing products. However, they potentially offer aspects of sustainability and energy efficiency which are in the order of the day.

## Conflict of interest statement

The author declares that the research was conducted in the absence of any commercial or financial relationships that could be construed as a potential conflict of interest.
